# Acknowledgments

**DOI:** 10.1002/ags3.12648

**Published:** 2023-01-10

The publication of invaluable papers in *Annals of Gastroenterological Surgery* depends on the prompt, careful review of submitted manuscripts. We would like to thank the following experts for reviewing manuscripts submitted between December 1, 2021 and November 30, 2022.

Abe, Tetsuya

Aizawa, Masaki

Ajiki, Tetsuo

Akagi, Yoshito

Akai, Hiroyuki

Akamatsu, Nobuhisa

Akita, Hirofumi

Akiyoshi, Takashi

Aoki, Takeshi

Aoki, Taku

Aoyama, Toru

Arigami, Takaaki

Ariizumi, Shun‐Ichi

Arita, Junichi

Ashida, Ryo

Baba, Yoshifumi

Ban, Daisuke

Bando, Etsuro

Beppu, Toru

Booka, Eisuke

Daiko, Hiroyuki

Deng, Han‐Yu

Ebata, Tomoki

Ebihara, Yuma

Eguchi, Hidetoshi

Etoh, Tsuyoshi

Fujii, Hodaka

Fujii, Tsutomu

Fujimura, Takashi

Fujishiro, Mitsuhiro

Fujita, Fumihiko

Fujita, Takeo

Fujiwara, Hitoshi

Fujiwara, Yoshiyuki

Fukami, Yasuyuki

Fukumoto, Takumi

Fukuyo, Ryosuke

Furuhata, Tomohisa

Goi, Takanori

Goto, Yuichi

Gotohda, Naoto

Hachisuka, Takehiro

Harimoto, Norifumi

Hasegawa, Hirotoshi

Hasegawa, Kiyoshi

Hasegawa, Suguru

Hasegawa, Yasushi

Hashimoto, Daisuke

Hata, Koichiro

Hata, Taishi

Hatano, Etsuro

Hatori, Takashi

Hayashi, Yoshito

Hazama, Shoichi

Hibi, Taizo

Hida, Koya

Hidaka, Masaaki

Higuchi, Ryota

Hiraki, Yoshiki

Hirano, Satoshi

Hirata, Keiji

Hiyoshi, Yukiharu

Honda, Goro

Hoshino, Isamu

Hosoda, Kei

Ikeda, Masataka

Ikegami, Toru

Ikeuchi, Hiroki

Ikoma, Hisashi

Inaki, Noriyuki

Inoue, Tadahisa

Isaji, Shuji

Ishido, Keinosuke

Ishihara, Soichiro

Ishii, Yoshiyuki

Ishizawa, Takeaki

Itabashi, Michio

Itano, Osamu

Itoh, Shinji

Kaibori, Masaki

Kaido, Toshimi

Kajiwara, Yoshiki

Kamatani, Takashi

Kamei, Takashi

Kanaji, Shingo

Kanda, Mitsuro

Kanemitsu, Yukihide

Kasama, Kazunori

Katai, Hitoshi

Kato, Yutaro

Kawada, Kenji

Kawai, Kazushige

Kawai, Manabu

Kawaida, Hiromichi

Kimura, Yasutoshi

Kimura, Yutaka

Kinami, Shinichi

Kinoshita, Takahiro

Kinugasa, Yuske

Kishi, Yoji

Kitago, Minoru

Kitayama, Joji

Kobayashi, Hirotoshi

Kobayashi, Shogo

Kojima, Kazuyuki

Komatsu, Shuhei

Komori, Koji

Konishi, Tsuyoshi

Kono, Koji

Kubo, Shoji

Kubota, Takeshi

Kudo, Atsushi

Kumamoto, Kensuke

Kunisaki, Chikara

Kuriu, Yoshiaki

Kuroda, Shinji

Lee, Han Hong

Makino, Isamu

Makino, Tomoki

Makiura, Daisuke

Manabe, Tatsuya

Marubashi, Shigeru

Matsuda, Chu

Matsuda, Keiji

Matsuda, Kenji

Matsuhashi, Nobuhisa

Matsui, Junichi

Matsumoto, Ippei

Matsumoto, Sohei

Matsuoka, Tadashi

Matsutani, Takeshi

Matsuyama, Ryusei

Mima, Kosuke

Mine, Shinji

Misawa, Takeyuki

Miura, Fumihiko

Miyamoto, Yuji

Miyazaki, Yasuhiro

Mizuguchi, Toru

Mizuno, Shugo

Mizuno, Takashi

Monden, Kazuteru

Mori, Shinichirou

Mori, Toshiyuki

Morikawa, Takanori

Morine, Yuji

Morise, Zenichi

Morita, Masaru

Motoi, Fuyuhiko

Motoori, Masaaki

Motoyama, Satoru

Murata, Kohei

Nagakawa, Yuichi

Naitoh, Takeshi

Nakagawa, Kei

Nakajima, Masanobu

Nakamura, Masafumi

Nakamura, Yoshiharu

Namikawa, Tsutomu

Nanashima, Atsushi

Niihara, Masahiro

Ninomiya, Itasu

Nishiwada, Satoshi

Nitta, Hiroyuki

Noda, Takehiro

Noma, Kazuhiro

Nomura, Sachiyo

Noshiro, Hirokazu

Nozawa, Hiroaki

Nunobe, Souya

Obama, Kazutaka

Obara, Hideaki

Ochiai, Toshiya

Oda, Tatsuya

Ohashi, Manabu

Ohta, Masayuki

Ohtsuka, Masayuki

Ohtsuka, Takao

Ojima, Toshiyasu

Okabayashi, Koji

Okabayashi, Takehiro

Okamoto, Kohji

Okamoto, Koichi

Okamura, Yukiyasu

Okano, Keiichi

Okita, Kenji

Ono, Hiroaki

Ono, Satoshi

Oshikiri, Taro

Saeki, Hiroshi

Sakamoto, Yoshihiro

Sano, Tsuyoshi

Sasaki, Akira

Satoi, Sohei

Seki, Yosuke

Seo, Satoru

Shichinohe, Toshiaki

Shida, Dai

Shimada, Yoshifumi

Shimizu, Yasuhiro

Shindo, Yoshitaro

Shinoda, Masahiro

Shinto, Eiji

Shiozaki, Atsushi

Soejima, Yuji

Suda, Koichi

Sugiura, Teiichi

Suzuki, Hiromu

Suzuki, Kenji

Suzuki, Nobuaki

Tajima, Yoshitsugu

Takada, Yasutsugu

Takahara, Takeshi

Takahashi, Hidenori

Takahashi, Takao

Takahashi, Tsuyoshi

Takatsuki, Mitsuhisa

Taketomi, Akinobu

Tanaka, Hiroaki

Tanaka, Keitaro

Tanaka, Koji

Tanaka, Kuniya

Tani, Masaji

Tatsuo, Tajiri

Taura, Kojiro

Terashima, Masanori

Toiyama, Yuji

Tokumitsu, Yukio

Tokunaga, Masanori

Tomimaru, Yoshito

Tsukamoto, Shunsuke

Tsunoda, Shigeru

Uchida, Yoichiro

Uemura, Kenichiro

Uetake, Hiroyuki

Umeda, Yuzo

Wada, Hiroshi

Wakai, Toshifumi

Wakasugi, Masaki

Wakiya, Taiichi

Watanabe, Manabu

Watanabe, Masayuki

Yamada, Suguru

Yamaguchi, Shigeki

Yamaguchi, Tatsuro

Yamamoto, Hirofumi

Yamamoto, Kazuyoshi

Yamamoto, Seiichiro

Yamamoto, Takehito

Yamashita, Keishi

Yamashita, Kotaro

Yamashita, Yo‐Ichi

Yasuda, Takushi

Yasui, Masayoshi

Yokoyama, Yukihiro

Yoshida, Hiroshi

Yoshida, Masashi

Yoshida, Naoya

Yoshimatsu, Kazuhiko

Yoshitomi, Hideyuki

Yoshizumi, Tomoharu

